# Examining subgroup effects by socioeconomic status of public health interventions targeting multiple risk behaviour in adolescence

**DOI:** 10.1186/s12889-018-6042-0

**Published:** 2018-10-16

**Authors:** Laura Tinner, Deborah Caldwell, Matthew Hickman, Georgina J MacArthur, Denise Gottfredson, Alberto Lana Perez, D Paul Moberg, David Wolfe, Rona Campbell

**Affiliations:** 1 Population Health Sciences, Bristol Medical School, University of Bristol, Canygne Hall, Bristol, BS8 2BN UK; 2 Department of Criminology and Criminal Justice, University of Maryland, College Park, Prince George’s, MD USA; 3 Department of Preventive Medicine and Public Health, School of Medicine and Health Sciences, University of Oviedo, Oviedo, Spain; 4 Department of Population Health Sciences, University of Wisconsin School of Medicine and Public Health, Madison, WI USA; 5 Faculty of Education, Western University, Ontario, Canada

**Keywords:** Interventions, Inequalities, Socioeconomic status, SES, Multiple risk behaviour, Adolescence, Systematic review

## Abstract

**Background:**

Multiple risk behaviour (MRB) refers to two or more risk behaviours such as smoking, drinking alcohol, poor diet and unsafe sex. Such behaviours are known to co-occur in adolescence. It is unknown whether MRB interventions are equally effective for young people of low and high socioeconomic status (SES). There is a need to examine these effects to determine whether MRB interventions have the potential to narrow or widen inequalities.

**Methods:**

Two Cochrane systematic reviews that examined interventions to reduce adolescent MRB were screened to identify universal interventions that reported SES. Study authors were contacted, and outcome data stratified by SES and intervention status were requested. Risk behaviour outcomes alcohol use, smoking, drug use, unsafe sex, overweight/obesity, sedentarism, peer violence and dating violence were examined in random effects meta-analyses and subgroup analyses conducted to explore differences between high SES and low SES adolescents.

**Results:**

Of 49 studies reporting universal interventions, only 16 also reported having measured SES. Of these 16 studies, four study authors provided data sufficient for subgroup analysis. There was no evidence of subgroup differences for any of the outcomes. For alcohol use, the direction of effect was the same for both the high SES group (RR 1.26, 95% CI: 0.96, 1.65, *p* = 0.09) and low SES group (RR 1.14, 95% CI: 0.98, 1.32, *p* = 0.08). The direction of effect was different for smoking behaviour in favour of the low SES group (RR 0.83, 95% CI: 0.66, 1.03, *p* = 0.09) versus the high SES group (RR 1.16, 95% CI: 0.82, 1.63, *p* = 0.39). For drug use, the direction of effect was the same for both the high SES group (RR 1.29, 95% CI: 0.97, 1.73, *p* = 0.08) and the low SES group (RR 1.28, 95% CI: 0.84, 1.96, *p* = 0.25).

**Conclusions:**

The majority of studies identified did not report having measured SES. There was no evidence of subgroup difference for all outcomes analysed among the four included studies. There is a need for routine reporting of demographic information within studies so that stronger evidence of effect by SES can be demonstrated and that interventions can be evaluated for their impact on health inequalities.

## Background

Risk behaviours such as smoking, alcohol consumption, drug misuse, risky sexual behaviour, unhealthy diet and low levels of physical activity co-occur in adolescence [1, 2]. Multiple risk behaviour (MRB) refers to the occurrence of two or more risk behaviours directly or indirectly related to health [3]. Recognising the co-occurrence of risk behaviours, there has been a number of public health interventions that address MRB as opposed to single behaviours in isolation [4]. The rationale behind this approach being that strategies may affect more than one outcome, proving more efficient and cost-effective [5]. Interventions targeting one behaviour may be less successful as they do not address the co-occurrence of behaviours [6]. The fact that most adults claim to have initiated risk behaviours during adolescence indicates that intervening early may be the best approach [7].

The inverse relationship between risk behaviour and socioeconomic status (SES) has been widely reported [8–10]. In response, strategies to improve health behaviour of young people have often targeted low SES groups. However, targeting ignores the social gradient to health and may result in stigma or failing to reach at-risk schools or pupils [11]. Thus, some researchers argue for ‘proportionate universalism’ [12] as “universal interventions which disproportionally benefit low SES groups may have the greatest potential to improve population health, while reducing inequality” [11]. However, proportionate universalism has potential challenges that have not been tested in practise, such as assessing the proportion of resource that should be allocated to each level of disadvantage [13].

It has been raised by a number of researchers, including those working in the Cochrane Equity Methods Group [14, 15], that universal public health interventions have the potential to increase inequalities in the population [15–17]. This occurs when the interventions are of greater benefit to the most advantaged groups, inadvertently increasing inequalities (the inverse equity hypothesis) [17, 18]. White et al. [19] state that all processes in the planning and delivery of public health strategies have the potential to create intervention generated inequalities. For instance, if a survey is used to assess need for a public health intervention, variation in response rates by socioeconomic status could underestimate the need among the most disadvantaged groups [19, 20]. Compliance with the intervention programme may also be higher in groups with higher SES who have greater access to resources such as time, coping skills and finance that enable them to take advantage of the intervention [16].

There are increasing initiatives to encourage more comprehensive reporting of factors that can lead to health inequalities [17]. PROGRESS-Plus is a proposed framework for the PRISMA Equity Extension, used to identify characteristics that stratify health opportunities and outcomes [17]. The acronym refers to the following: place of residence; race/ethnicity/culture/language; occupation; gender/sex; religion; education; socioeconomic status and social capital, with the ‘Plus’ element referring to personal discriminating characteristics (e.g. age, disability), features of relationships (e.g. smoking parents) and time-dependent relationships (e.g. leaving the hospital, temporary disadvantage) [17]. Although this framework is now widely adopted and has been endorsed by the Campbell and Cochrane Equity Methods Group [21], there is still limited evidence about intervention generated inequalities from systematic reviews. This is in part due to the studies under review failing to record such demographic information in the first instance [22].

Whereas reviews have been undertaken to assess inequalities with regard to single behaviours such as healthy eating [16, 22] physical activity [23], smoking tobacco [24], no study has yet explored differential intervention effects with regard to MRB interventions. This study re-examines studies from two Cochrane systematic reviews of adolescent MRB interventions [4, 6] for differential outcome effects by socioeconomic status. The aim was to identify whether public health interventions for adolescent MRB increase or reduce inequalities. It was also an aim of the study to determine the extent to which SES is reported within adolescent MRB studies and the types of measures used.

## Methods

### Study design

We conducted secondary analyses of studies included in two Cochrane systematic reviews that focused on adolescent MRB [4, 6]. These reviews included randomised controlled trails (RCTs) and cluster RCTs. The reviews examined the effects of interventions implemented up to 18 years of age for the primary or secondary prevention of multiple risk behaviours. The interventions could be individual, family, or school-based interventions. Risk behaviours included: tobacco use; alcohol consumption; illicit drug use; risky sexual behaviours; anti-social behaviour and offending; vehicle-related risk behaviours; self-harm; gambling; unhealthy diet; high levels of sedentary behaviour; and low levels of physical activity. Both reviews followed the robust procedures specified by the Cochrane Collaboration and the interested reader is referred to the protocols for further details [4, 6]. The literature searches for these reviews were conducted in 2012 and 2015.

### Inclusion criteria and screening for current analysis

Studies were eligible for the current paper if they met the initial inclusion criteria for the Cochrane reviews on adolescent MRB [4, 6]. Additionally, studies were restricted to universal interventions (i.e. aimed at a whole population, such as a school) [11], as interventions that target specific demographic or high-risk groups would be unable to detect difference in effect by SES. If a study was described as universal, but participants were predominantly from one socioeconomic group, it was excluded from this analysis. Full text studies were then screened to determine if any reported having measured SES at baseline and/or conducted a subgroup analysis by SES. SES is broadly defined to include a range of indicators such as: parental education, parental income, parental occupation, free school meal eligibility, Hollingshead index [25] and receipt of social benefit. Other demographic indicators outlined by the PROGRESS-Plus [17] model such as gender and ethnicity were not included in this analysis.

### Data extraction and management

SES data were extracted by one reviewer and input into a predesigned form; additional data on intervention design, setting, population, and outcome(s) were taken from the Characteristics of Studies tables produced for [4, 6]. Authors of the study papers that reported having measured SES at baseline were contacted and asked to provide additional detail on the outcomes by SES and intervention assignment (i.e. experimental or control). Authors were also given the option to send the cleaned dataset if they preferred. Those contacted were given several weeks to respond and reminder emails were sent.

### Data analysis

Following the procedure of project TEENAGE [26], SES measures were dichotomized into high SES (parent with degree-level education, not eligible for free school meals) and low SES (parent without a degree-level education, eligible for free school meals). Studies were combined using a random effects meta-analysis and sub-group analyses performed using Revman 5.3 (The Nordic Cochrane Centre, The Cochrane Collaboration, Copenhagen 2014). Random-effects models were specified a priori due to anticipated between-study heterogeneity with regard to settings and participants [27]. Subgroup-specific effect estimates are summarised as relative risks (RR) with 95% confidence intervals (CI) and are presented in forest plots by dichotomised SES subgroup. We do not report the overall pooled summary effect (i.e. the two subgroups combined) since the studies included here are a subset of those included in the original Cochrane reviews. An overall pooled effect would therefore be misleading. Subgroup-specific intervention effects are compared using a test for interaction rather than comparison of significance through *p*-values [27]. The test for interaction is undertaken using Cochran’s Q and Higgins I^2^. [28, 29]. Outcomes only examined by a single study, are reported in the text.

## Results

### Description of included studies

**Fig. 1:**
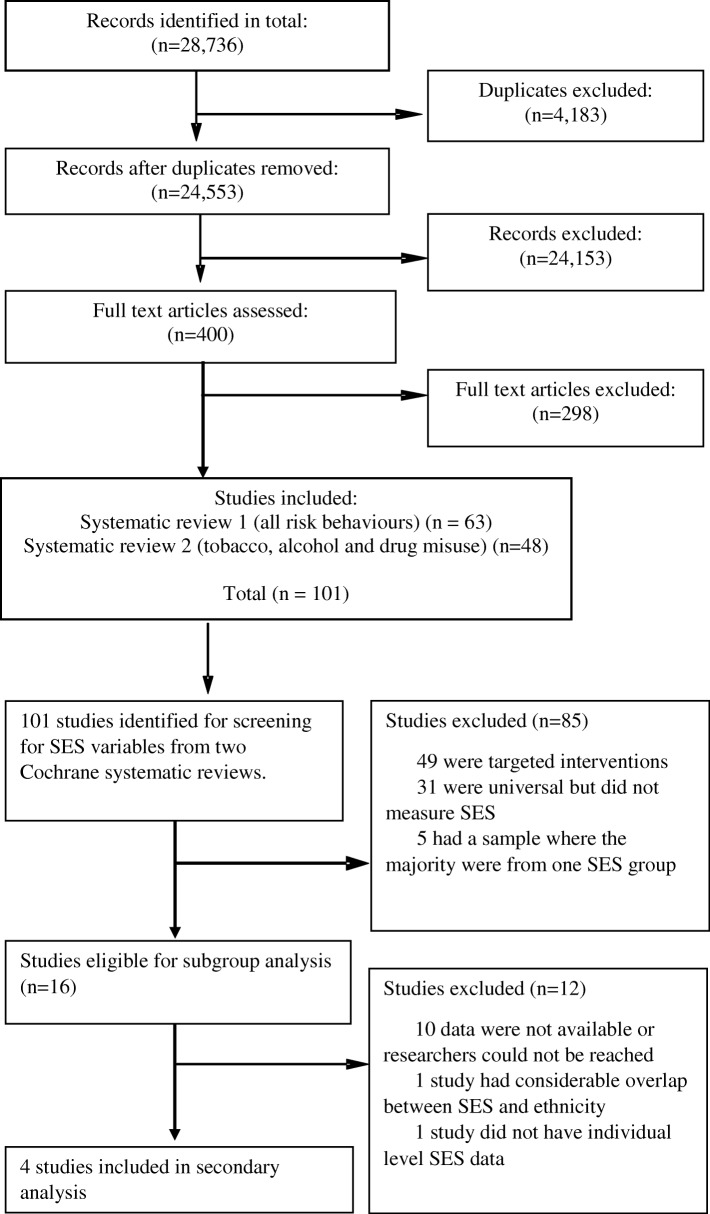
Flow diagram detailing the systematic review screening process. The figure shows at what stage studies were excluded from the eligible dataset and gives reasons for exclusion, resulting in the final sample

Figure 1 presents the flow chart of included studies which, for transparency, includes the screening process for the ancestor Cochrane systematic reviews. Following the full text screening of 101 eligible studies (published between 1982 and 2015) from the parent Cochrane reviews, 49 studies met the criterion of being universal and not targeted to a specific group. Of those universal studies, 16 reported measuring SES and were therefore eligible for secondary analysis for the current paper and authors were contacted for additional data on SES. These studies are included in Table 1, along with descriptive information on: study name, setting, population, intervention properties, primary outcome(s), SES indicator used, effect of intervention, differential effect by SES and risk of bias assessments. Studies were conducted in the following countries: USA (*n* = 12), UK (*n* = 1), Canada (*n* = 1), Spain and Mexico collaboration (*n* = 1) and Sweden (*n* = 1). Of the 16 eligible studies, the following types of intervention were described, with some studies adopting more than one intervention element: school-based curriculum, workshop, training or problem solving (*n* = 13), family or parent training or support (*n* = 5), adult mentoring programme (*n* = 1), website and text service (*n* = 1), practice nurse session (*n* = 1). Details and reasons for study exclusion are listed in Table 2 and Table 3 in Appendix 1 and 2.

**Table 1:** Descriptive characteristics of studies eligible for secondary analysis

Study	Setting	Population	Intervention	Primary outcome(s)	SES measure at baseline	Effect	Risk of Bias
Bodin and Leifman 2011 [54]	Community, Sweden	128 recruited, 65 to intervention, 63 to control	Adult mentoring over the course of one year	Alcohol use Drunk in the last month Delinquency Substance use in the last 6 months Tobacco user Depressive symptoms	Parental degree	No significant outcome differences between the groups. Low statistical power preclude definite conclusions.	
*Raising Healthy Children* Brown et al 2005 [55]	Schools in Washington, USA	1,040 students from 10 schools, 5 schools to intervention and 5 to control.	Prevention strategies that address risk and protective factors that consisted of teacher development workshops, student after-school tutoring sessions and parenting workshops for families.	Substance use: alcohol, marijuana and tobacco use.	Household income status	There were significant intervention effects in growth trajectories for frequency of alcohol and marijuana use but not for use versus non-use.	
*Family Check-Up* Connell et al 2007 [56]	Middle Schools, Oregon USA	998 recruited from 3 middle schools, 500 to intervention 498 to control	Universal classroom-based intervention, the Family Check-Up and family management treatment	Adolescent substance use: tobacco, alcohol, drugs Problem behaviour: antisocial behaviour and offending	Free school meal eligibility	Relative to controls, adolescents whose parents engaged in the Family Check-Up exhibited less growth in alcohol, tobacco, and marijuana use and problem behaviour during ages 11 through 17. There was also a decreased risk for substance use and arrests by age 18.	
*DARE-A* and *RSTP* D'Amico 2002 [57]	One high school, USA	300 recruited from 1 school. 75 to intervention arm 1, 75 to intervention arm 2, 150 control.	Risk skills training programme interactive group session and DARE-A drug abuse and resistance education programme.	Tobacco Alcohol use Marijuana use Violence Victimisation	Family income	RSTP participants decreased participation in several risk behaviours as post-test, but reductions were not maintained at 6-month follow-up. Both the control and the DARE-A groups decreased negative alcohol expectancies and the control group increased alcohol consumption.	
*LIFT* DeGarmo et al 2009 [58]	Schools in North West USA	671 recruited from 12 schools	Parent management training, child social and problem solving skills and school recess component	Alcohol use Cannabis use Tobacco use Arrests	Annual household income	LIFT had a significant effect on reducing the rate of growth in use of tobacco, alcohol and illicit drugs. Average tobacco use reductions were mediated by increases in family problem solving.	
*All Stars* Gottfredson et al 2010 [43] (included in subgroup analysis)	Schools in Maryland, USA	447 from 5 schools. 224 participants to intervention 223 to control	All Stars programme with lessons about substance use prevention, violence prevention and “plus” lessons reinforcing attitudes to behaviour change. Programme delivered in an after school setting.	Alcohol initiation Tobacco initiation Marijuana initiation Drug use in the past 30 days School attendance	Free school meal eligibility, Parent income	Results show no difference between the treatment and control students and post-test at any outcomes or mediators. No positive effects were found for youths receiving higher dosage.	
*Parents who care* Haggerty et al 2007 [59]	Seattle, USA	331 youths and parents, 107 to intervention 1, 118 to intervention 2 and 106 to control	SA group - 10 week programme including video and workbook, family consultant contact by phone.	Violence in the past 30 days Alcohol use Initiation of sex Illegal drug use Marijuana use Cigarette use	Household income and parental education	No intervention effect was found on rate of change in attitudes about drug use or frequency of delinquent behaviour. Regression analysis with multiple imputation found a reduction in favourable attitudes to drug use and significantly less violent behaviour than the controls. No effects were found for drug use or delinquency. Both intervention groups were found to be less likely to initiate substance use and/or sexual activity than those in the control arm.	
*Keepin’ it Real* Hecht et al 2003 [59]	Elementary schools, USA	1,556 students from 23 schools. 10 schools to intervention and 13 schools to control.	Originally an intervention for 7^th^ grade students adapted for young students in 5^th^ grade. The program consists of 10, 45-minute lessons which incorporate 5 videos and content on enhancing anti-drug expectancies, normative beliefs and refusal self-efficacy and facilitating decision-making and resistance skills.	Tobacco use Alcohol use Marijuana use	Free school meal eligibility	The intervention generally appeared no more effective than the control schools’ programming in changing students’ resistance or decision-making skills; substance use intentions, expectations, normative beliefs or life time and recent substance use.	
*Family Schools Partnership and Classroom Centred* Ialango et al 1999 [60]	Primary schools Maryland, USA	230 to CC, 229 to FSP and 219 to control	CC- Curriculum enhancement and behaviour management and weekly meeting to promote problem solving skills. FSP – training for teachers in parent communication, weekly home-school learning, nine workshops and a school psychologist.	Aggressive and shy behaviour Substance use Affective disorder Conduct disorder	Free school meal eligibility	CC and FSP participants were found to have significantly fewer problem behaviours than control participants as rated by teachers.	
*Good Behaviour Game* Kellam et al 2014 [61]	Primary Schools in Maryland, USA	1196 from 19 schools. 238 to intervention 169 to control.	Classroom team-based behaviour management strategy. Children assigned to teams and rewarded for good behaviour.	Alcohol abuse Drug use Smoking High school graduation Condom use	Free/reduced school meal status	By young adulthood significant impact was found among males in intervention group in reduced drug and alcohol abuse, regular smoking and antisocial personality disorder.	
*Prevencanadol* Lana et al 2014 [31] (included in subgroup analysis)	Secondary schools in Spain and Mexico	2001, 1014 to intervention 987 to control	Website to learn how to prevent and treat main cancer risk behaviours. Weekly texts to encourage compliance with healthy behaviours.	Smoking Dietary fat Alcohol Sedentarism Not enough fruits Overweight/obesity	Parental degree	At 9 month follow up, the prevalence of students who did not eat fruit reduced significantly in both intervention groups. Being overweight reduced in intervention group 2. Overall cancer behavioural risk score decreased in both intervention groups.	
*Michigan Model for Health* O’Neill et al 2011 [62]	Schools in Michigan and Indiana, USA	2512 from 52 schools. 1345 in intervention schools, 1167 in control schools.	52 lessons delivered over a 2-year period. Content included social and emotional health, alcohol, tobacco, other drugs, safety, nutrition and physical activity.	Social and Emotional Skills Drug refusal skills Alcohol use Anti-social behaviour Tobacco use	Proportion of school eligible for free/reduced school meal	Students in the intervention group had better interpersonal communication skills, social and emotional skills and drug refusal skills than control students. Intervention students reported lower intentions to use alcohol and tobacco and less alcohol and tobacco use initiated in the last 30 days, as well as reduced levels of aggression.	
*Healthy for Life* Piper et al 2000 [63] (included in subgroup analysis)	Secondary schools, Wisconsin USA	2,483 from 21 schools, 827 to age appropriate intervention 758 to intensive intervention and 898 to control.	54-lesson curriculum delivered in either an intensive twelve week block or in three four-week segments (age appropriate). Use of peer leaders, parent-adult interviews, parent orientation, health advocacy, students making public commitments to health behaviours.	Nutrition Alcohol use Sexual intercourse Marijuana use Tobacco use	Maternal education	The intervention had minimal effect on participating students relative to control schools. The Intensive version was more effective than the Age Appropriate version, with small positive results on four measures (frequency of meals, perceptions of peer use, cigarettes and marijuana) and small negative effects on alcohol use.	
*Life Skills Training plus Strengthening Families Program: For Parents and Youth 10-14.* Spoth et al 2008 [64]	Schools in Midwest USA	1677 students from 36 schools. 543 students were assigned to intervention 1 which combine two elements (LST + SFP 10-14), 622 were assigned to LST only and 489 went to the control group.	Together the two intervention programmes targeted family, individual, school and peer related factors associated with adolescent substance use. The intervention is a 15-session classroom based programme (LST) based on social learning theory. The primary goal is to promote skill development concerning the avoidance of substance use. The additional 7-session SFP: 10-10 program targets factors in the family environment with goals of enhancing parenting skills as well as peer resistance skills.	Alcohol use Marijuana use Tobacco use	Free/reduced school lunch	For all substance initiation outcomes one or both the intervention groups showed significant differences at 12^th^ grade and growth trajectory outcomes compared with the control group.	
Walker et al 2002 [65]	General Practice Surgeries, Hertfordshire UK	1488, 746 to intervention and 742 to control	20 minute discussion with a practice nurse making plans to live a healthier lifestyle.	Alcohol use Physical activity Tobacco use Unhealthy diet	Parental occupation	More intervention students reported positive change for diet and exercise and at least one of four behaviours (diet, exercise, smoking, drinking alcohol) at 3-month follow-up, but this did not persist at 12 months.	
*Fourth R: Skills for Youth Relationships* Wolfe et al 2012 [30] (iB.)	Secondary schools, Ontario, Canada	1722 from 10 schools. 968 to intervention 754 in control.	Program taught in place of existing health curriculum. A 21 lesson curriculum delivered by teachers with specialism in health and physical education.	Physical dating violence Physical peer violence Condom use Problem substance use	Parental education	Physical dating violence was greater in control students than intervention students. The intervention effect was greater in boys than girls. The main effects for other outcomes did not have statistically strong evidence. However, boys who received the intervention show in a significant difference in condom use.	

Descriptive characteristics of studies eligible for secondary analysis including: Study name, author names and reference; setting; population; intervention; primary outcome(s); SES measure at baseline; effect; risk of bias assessment. Table 1 key for the risk of bias assessment:

Risk of bias assessment refers to the following seven domains, in the order they appear in the table, as instructed by Cochrane:

1. Random sequence generation (selection bias)

2. Allocation concealment (selection bias)

3. Blinding of participants and personnel (performance bias)

4. Blinding of outcome assessment (detection bias)

5. Incomplete outcome data (attrition bias)

6. Selective reporting (reporting bias)

7. Other bias

### Measures of SES

A range of SES measures was employed across the studies, with some using more than one SES measure. The most commonly used measure of SES was free school meal eligibility (*n* = 7), with one study also including reduced-price school meal eligibility. Parental education (*n* = 5) and parental/household income (*n* = 5) were the second most common, with one study specifying maternal education. None of the studies gave a rationale for their choice of SES measure.

### Subgroup analyses by SES

Of the 16 authors contacted for further data, nine authors did not respond and three did not have the data available in a suitable format to re-run analyses. Consequently, only four studies could be included in the secondary analysis. Of these, two were conducted in the USA, one in Canada and one as a collaboration between Spain and Mexico. All were school-based. All four studies measured alcohol use as a primary outcome, with other outcomes including tobacco smoking (*n* = 3), drug use (*n* = 3) with two studies measuring any illicit drug use and one measuring cannabis use, unsafe sex (*n* = 1), overweight/obese (*n* = 1), sedentarism (*n* = 1) dating violence (*n* = 1) and peer violence (*n* = 1). Two studies measured SES using free school meal eligibility and two used parental education as a proxy for adolescent SES. For alcohol, measured by all studies, the total number of participants in the subgroup analysis was 1720 in the high SES group and 1657 in the low SES group.

**Fig. 2:**
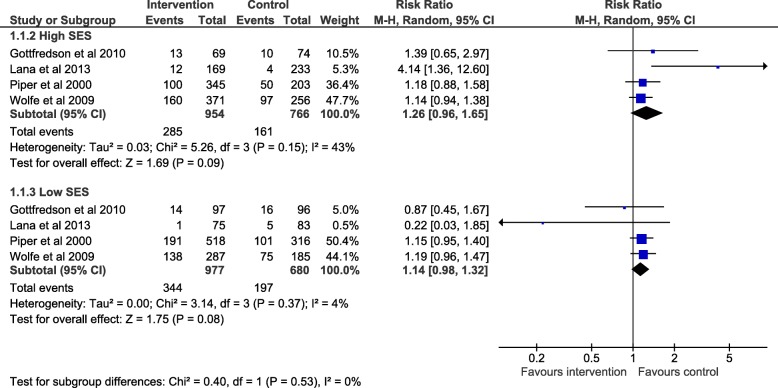
Forest plot of meta regression analysis for outcome alcohol by SES group. The plot shows the data meta-analysed in two subgroups (high SES: parent with a degree or ineligible for free school meals) (low SES: parents do not have a degree or young person eligible for free school meals). The boxes represent the estimates and the arrows coming out of the boxes the 95% confidence intervals. The diamond is the pooled estimate for each subgroup. The overall pooled estimate is not shown as the figure is concerned with comparing the two groups. Estimates on the right-hand side of the figure labelled ‘favours control’ equates to an increase in negative alcohol behaviour while the ‘favours intervention’ arm refers to a reduction in negative alcohol behaviour following intervention

**Fig. 3:**
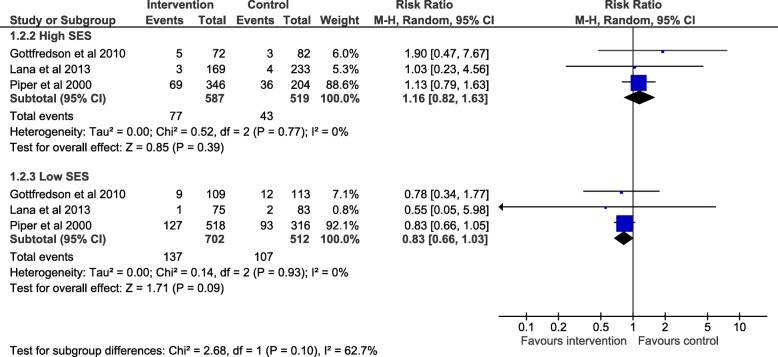
Forest plot of meta regression analysis for outcome smoking by SES group. The plot shows the data meta-analysed in two subgroups (high SES: parent with a degree or ineligible for free school meals) (low SES: parents do not have a degree or young person eligible for free school meals). The boxes represent the estimates and the arrows coming out of the boxes the 95% confidence intervals. The diamond is the pooled estimate for each subgroup. The overall pooled estimate is not shown as the figure is concerned with comparing the two groups. Estimates on the right-hand side of the figure labelled ‘favours control’ equates to an increase in smoking behaviour while the ‘favours intervention’ arm refers to a reduction in smoking behaviour following intervention

**Fig. 4:**
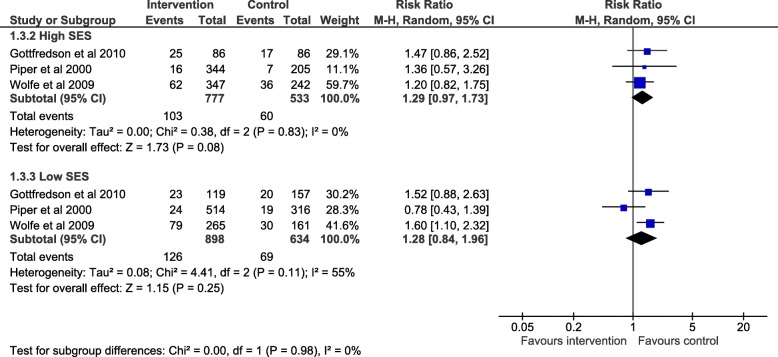
Forest plot of meta regression analysis for outcome drug use by SES group. The plot shows the data meta-analysed in two subgroups (high SES: parent with a degree or ineligible for free school meals) (low SES: parents do not have a degree or young person eligible for free school meals). The boxes represent the estimates and the arrows coming out of the boxes the 95% confidence intervals. The diamond is the pooled estimate for each subgroup. The overall pooled estimate is not shown as the figure is concerned with comparing the two groups. Estimates on the right-hand side of the figure labelled ‘favours control’ equates to an increase in drug use while the ‘favours intervention’ arm refers to a reduction in drug use following intervention

We report subgroup analyses in Fig. 2 for alcohol, Fig. 3 smoking and Fig. 4 drug use. The maximum number of studies included is 4. For all three outcomes there was an absence of evidence for subgroup differences by high or low SES: alcohol use (χ^2^ = 0.40, df = 1 (*p* = 0.53) I^2^ = 0%), smoking (χ2 = 2.68, df = 1 (*p* = 0.10) I2 = 62.7%) and drug use (χ2 = 0.00, df = 1 (*p* = 0.98) I2 = 0%). Due to the small numbers of studies included, these results should be interpreted with caution and may represent false negatives due to inadequate power.

The point estimates for alcohol use show that SES does not explain the effect of the intervention, as the direction of effect is the same for both high SES (RR 1.26, 95% CI: 0.96, 1.65, *p* = 0.09) and low SES (RR 1.14, 95% CI: 0.98, 1.32, *p* = 0.08). The confidence intervals for both groups’ pooled estimates cross the null.

For smoking the point estimates are indicative of a differential intervention effect in favour of the low SES group (RR 0.83, 95% CI: 0.66, 1.03, p = 0.09) versus the high SES group (RR 1.16, 95% CI: 0.82, 1.63, *p* = 0.39). The confidence intervals in both groups crossed the null.

For drug use, SES was not an explanatory factor for the intervention effect as the direction of effect in the high SES group (RR 1.29, 95% CI: 0.97, 1.73, p = 0.08) and the low SES group (RR 1.28, 95% CI: 0.84, 1.96, *p* = 0.25) was the same. The confidence intervals in both groups crossed the null.

Only 1 study reported outcomes related to peer and dating violence [30]. Analysis revealed no evidence of a difference between the SES groups within the individual study for peer violence (χ2 = 2.37, df = 2, *p* = 0.31, I2 = 15.6%) or dating violence (χ2 = 1.91, df = 2, *p* = 0.38, I2 = 0%). The point estimates suggested a beneficial intervention effect regarding dating violence for high SES young people (RR 0.49, 95% CI: 0.28, 0.86, *p* = 0.01) and an insufficient intervention effect for low SES adolescents (RR 0.84, 95% CI: 0.50, 1.14, *p* = 0.51). The direction of effect was the same for peer violence in Wolfe et al.’s [30] study: high SES adolescents (RR 0.85, 95% CI: 0.58, 1.25, *p* = 0.40) and low SES adolescents (RR 1.32, 95% CI: 0.88, 1.99, *p* = 0.18).

Only 1 study reported the outcome overweight/obesity [31]. The test for subgroup differences revealed no difference between the SES groups within the individual study (χ2 = 0.98, df = 2, *p* = 0.61, I2 = 0%). The point estimates suggested a differential intervention effect in favour of the low SES group (RR 0.87, 95% CI: 0.42, 1.80, *p* = 0.71). For sedentarism, examined in the same isolated study, the point estimates indicated that SES did not explain difference in the intervention effect as both were in the same direct: high SES (RR 0.77, 95% CI: 0.55, 1.07, *p* = 0.12) low SES: (RR 0.82, 95% CI: 0.47, 1.41, *p* = 0.47). The confidence intervals for the effect in both groups crossed the null.

## Discussion

Through a secondary analysis of intervention effects by socioeconomic status in adolescent MRB interventions, we found there to be sparse reporting of demographic characteristics needed to investigate inequalities.

Despite the recognition that interventions may increase or reduce inequalities, concerns have been raised that most evaluations do not assess the differential impact by socioeconomic group [11, 32]. Many studies do not record the demographic information needed for subgroup analysis, or are insufficiently powered [16, 22, 33]. Moore et al. [11] suggest that this inattention to inequality within public health interventions may reflect a utilitarian perspective in focusing on achieving the greatest benefit for the most amount of people. Lynch et al. [34] address this idea through a discussion of ‘absolute’ and ‘relative’ inequality. They note that if the aim is to reduce relative inequalities in an outcome, the absolute benefit to the population may be small, particularly if the outcome is equally common in all groups [34]. Therefore, for some outcomes there may be a conflict in attempting to improve health for all young people while also reducing inequalities.

There was variation in how SES is measured in the eligible studies, with no justification for the choice of measure in any of the full text papers [11]. This study supports Moore et al. [11] in illustrating the need for greater consensus upon SES measures to ensure more reliable studies. Even those studies that used the same SES measure may have employed it slightly differently, for example, only measuring mother or father’s education or including reduced-priced school meal eligibility as well as free school meals. A consensus on a single SES measure, however, may not be easily reached due to significant disagreements on definitions and theoretical assumptions [35]. For example, the most common measure in the eligible studies, free school meal eligibility, is often used a proxy of SES as it is easily accessible and inexpensive to record compared with parental education and household income [35]. However, the threshold for eligibility may differ between countries and regions and some authors have found free school meal eligibility to inadequately reflect household income [35, 36].

The Family Influence Scale (FAS) has been implemented in the Health Behaviour in School-Aged Children (HBSC) study, as well as other research and policy work as a measure of SES within adolescence [37]. FAS is comprised of easily answerable indicators of affluence and consumption, such as number of cars, young people having their own bedroom and the number of holidays the family take. Studies adopting FAS have reported low levels of missing data of around 2% [37–39] and have shown low affluence to be associated with outcomes such as increased risk of fighting injury [40] and higher consumption soft drinks compared to high affluence young people [41]. FAS thus presents a possible indicator for assessing inequalities in interventions that target adolescents. Although, there are limitations such as the need to adapt the scale dependent on country and consumer patterns [37] and most validation work has been done within cross-sectional studies [37].

The point estimates for alcohol use show that SES does not explain the effect of the intervention as the direction of effect is the same for both SES groups, indicating that these interventions neither increase nor reduce inequalities. However, there was a lack of evidence of subgroup difference, so these findings are indicative. The point estimates favoured the control group, which is noteworthy as the findings from the meta-analysis from one of the original MRB systematic reviews reported universal school-based interventions to have a beneficial effect for young people in relation to alcohol use [42]. One reason for this difference in findings could be that this present subgroup analyses had only four studies. Two of the four studies [31, 43] in the subgroup analysis had small samples and low numbers of events, meaning that the pooled estimates were heavily weighted toward the other two studies. The original systematic review from which these studies were taken, however, meta-analysed eight studies with 8751 participants in total [42] demonstrating that such interventions probably have a beneficial effect in relation to alcohol. Thus, it is further evident that there is a need for greater reporting of demographic characteristics so that health inequalities in interventions might be examined more comprehensively.

For tobacco smoking, there was an indication of a different direction of effect between the groups, in favour of the low SES group. The confidence intervals cross the null in these analyses and there is no evidence of subgroup differences. However, this finding highlights the potential for interventions to have differential effects for different groups. Reviews with more studies and greater power are needed to investigate this further, as the thinking that universal interventions tend to benefit those from high SES the most [11, 15] might not always be the case. The intervention effect on inequalities may depend on the setting, the intervention type and the outcomes being examined. A systematic review that assessed the equity impact of tobacco interventions and policies on young people found that price increase of cigarettes had the most consistent positive impact [44]. However, the review found very few studies that examined intervention inequalities among young people and called for the evidence base to be strengthened [44]. Therefore, there is a need for greater exploration of the types of interventions (upstream or downstream) and intervention elements that may decrease inequalities in MRB in young people.

For Wolfe et al.’s [30] study, the school-based training intervention, the direction of effect was different between groups for outcomes dating violence and peer violence, in favour of the high SES group. In the overall sample Wolfe et al. [45] reported a beneficial effect for both dating violence and peer violence. This finding represents one example of an intervention that is successful at a population level, yet may potentially increase inequalities through disproportionately benefiting the least deprived adolescents. The fact that the intervention under evaluation was a downstream intervention, focusing on individual behaviour change, chimes with current thinking around intervention generated inequalities [15, 46]. However, this example should be treated as illustrative as it only presents one study and there was no evidence of subgroup difference between the low SES and high SES groups.

This present study supports previous research in highlighting the limited evidence base on the differential effect of interventions by socioeconomic status. Researchers have cited similar difficulties in being able to find studies that conduct subgroup analyses, that measure socioeconomic variables at baseline or that are powered to detect differences by demographic groups [11, 15, 22, 46]. The TEENAGE project reanalysed interventions targeting any of four risk behaviours (smoking, diet, physical activity and alcohol) for differential effects by SES, but was limited by the small number of studies that collected demographic information [26, 47]. Moore et al. [11] conducted a systematic review of school-based health interventions but could only draw tentative conclusions due to a small sample and called for more routine testing of effects of interventions on inequality. While our study was also unable to detect evidence of difference due to a small sample, it provides an indication of how interventions may affect different SES groups differently.

This study highlights that work still needs to be done to encourage intervention leaders and systematic reviewers to record and analyse PROGRESS-Plus demographic information. The fact that only a small proportion of identified interventions measured SES at baseline, and none conducted SES sub-group analysis, further illuminates the lack of evidence regarding intervention generated inequalities [19]. This present study also highlights the importance of researchers keeping datasets accessible and being willing to engage in re-analysis.

### Strengths and limitations

A strength of this study is the systematic approach to reviewing public health interventions for differential outcome effect by SES. To our knowledge this is the first of its kind to conduct secondary analysis on universal interventions aimed at adolescent multiple risk behaviour using Cochrane systematic reviews. The approach could be applied for other public health interventions looking at different outcomes in different age groups. This study also demonstrates potential research that can be done to investigate health inequalities using existing systematic reviews at relatively low cost. It further highlights the need to consider health inequalities in the development of public health interventions so that appropriate subgroup analyses and meta-analyses are possible to detect differential effects by different SES groups.

Secondary analyses were only performed on a small sample of studies, most of which were not initially designed or sufficiently powered to investigate inequalities. Post hoc subgroup analyses can be unreliable and misleading [48]; however, not performing them would be a lost opportunity in terms of investigating health inequalities [46]. Most of the studies were conducted in North America, which makes generalizability difficult due to the different ways SES affects health cross-nationally [11, 46], for instance with regard to the racialisation of inequality in North America [11]. Many of the interventions included in the Cochrane systematic reviews were at least 10 years old so it is possible that the collection and reporting of demographic variables to trace intervention generated inequalities is occurring in more contemporary studies not captured by these reviews. We were unable to find any study protocols, so we are unaware if some studies intended to collect SES data but did not publish the findings. Therefore, selective outcome reporting could be a problem among the included studies [49].

There was considerable heterogeneity among the studies regarding design and statistical methodology. There was also variation in the type of SES measure used, making comparability a challenge [50].

Another limitation that has been cited in similar studies [46] is that all of the interventions re-analysed were ‘downstream’ interventions. Thus, we are unable to compare upstream and downstream interventions in their impact on health inequalities, which would be preferable as it has been hypothesized that ‘upstream interventions’ may be the most successful in reducing inequality [15]. Had we included other study designs, such as natural experiments, we may have been able to explore more upstream interventions as well as studies that specifically look at the effect of interventions on inequalities [51].

There was socially patterned attrition in some of the included studies, meaning that the low SES groups often had fewer participants than high SES groups. The behaviour outcome measures were also self-report, which may lead to less valid completion among some demographic groups and thus lack the sensitivity to detect differential effects [11, 52].

There is the additional measurement problem when studying inequalities in young people. If parental measures of SES are used as proxy and are self-report by their children, they may not know the educational attainment or occupation of their parents [37, 53]. It is likely there will be missing data that are socially patterned, with higher SES young people being more aware of their parents’ education and occupation than low SES young people [37].

## Conclusions

The majority of studies identified did not report having measured SES. There was little consistency of SES measure used and rarely were demographics reported for the purposes of subgroup analyses, meaning that this study was underpowered to detect subgroup differences. There is a need for routine reporting of demographic information within studies so that stronger evidence of effect by SES can be demonstrated and interventions evaluated for their impact on health inequalities.

## Appendix 1

**Table 2:** Excluded studies that target to a specific group. Targeted studies not included in the analysis and to which group the intervention was targeted towards

Primary Authors	Trial	Targeted to which group
Bernstein 2010 [66]	Reaching Adolescents for Prevention	Young people with high levels of risk behaviour
Berry 2009 [67]	Coaching for Communities	Young people with at least one of five key risk factors
Bond et al. 2001 [68]	Gatehouse	Government, independent and Catholic schools
Brody 2012a [69]	Adults in the making	Targeted to African American families in rural Georgia
Brody 2012b [69]	SAAF-T	Targeted to African American families in rural Georgia
Bush 1989 [70]	Know Your Body 2	Black students in Columbia district
Catalano 1999 [71]	Focus on Families	Parents in methadone treatment and their children
Clark et al. 2010 [72]	SUCCESS	Youth with behavioural problems
Conduct Problems Prevention Research Group 2014 [73]	Fast Track	Children with conduct problems
Cunningham 2012 [74]	SafERteens	Hazardous and harmful adolescent drinkers attending emergency department unit
Elder et al. 2002 [75]	Migrant education	Migrants - predominantly Mexican
Fang 2010 [76]	Mother-Daughter - Asian-American	Asian-American adolescent girls - second generation from socioeconomically advantaged backgrounds
Flay 2004 [77]	Aban Aya	Predominantly African-American schools, high risk sample
Freudenberg 2010 [78]	REAL MEN	Male participants recruited in prisons
Friedman 2002 [79]	Botvin LST and Anti-Violence	Inner-city, low SES, court adjudicated males convicted of at least one offence
Gersick 1988 [80]	Gersick	Public schools from predominantly working and lower -middle class towns
Gilchrist et al. 1987 [81]	Skills enchancement prog	American Indian youth
Gonzales [82]	Bridges to High School	Family program targeted to Mexican Americans
Griffin 2009 [83]	BRAVE	Inner city African-American majority
Hallfors et al. 2006 [84]	Reconnecting youth	Identified by school as being high risk - top 25% for truancy and bottom 50% for GPA or referral by school teacher/counsellor
Horan et al. 1982 [85]	Assertion training	Those with the lowest Assertive Behaviour Test (ABT) scores i.e. least assertive individuals
Kim 2011 [86]	Middle School Success	Girls in foster care, who are about to enter middle school
Kitzman 2010 [87]	Nurse Family Partnership 2	Females less than 29 weeks gestation, with socio-demographic risk characteristics
Komro et al. 2008 [88]	Project northland	Urban, multi-ethnic and low-income population
Lewis 2013 [89]	Positive Action (Chicago)	Undergraduates engaged in alcohol or sexual risk behaviour
Li 2002 [90]	imPACT	African-American parent-adolescent dyads
Lochman 2003 [91]	Coping Power 1	Targeted at children at risk of aggressive/disruptive behaviour
Lochman 2004 [92]	Coping Power 2	Targeted at boys at risk of aggressive or disruptive behaviour
LoSciuto 1999 [93]	Woodrock Youth Development Project	Deprived community, with large % of children from families receiving financial assistance from state
Marsden et al. 2006 [94]	Marsden	Users of ecstasy, crack cocaine or powder cocaine
McBride-Murray 2014 [95]	SAAF	Rural African American
McCambridge et al. 2011 [96]	Motivational interviewing 2011	Mainly those who had not completed Level 2 education (i.e. not achieved conventional measure of educational attainment on completion of compulsory schooling
Milburn 2012 [97]	STRIVE	Newly homeless youth and their families
Minnis 2014 [98]	Yo Puedo	Latino participants aged 16–21 years, residing in San Francisco
Monti 1999 [99]	Alcohol Screen and B.I. 1	Individuals aged 18–19 years, with drink-related emergency department attendances
Morris 2003 [100]	Self-Sufficiency Project	Targeted at low income single parent families from 2 Provinces in Canada
Newton et al. 2009 [101]	Climate schools	Independent (private) schools
Nirenberg 2013 [102]	ROAD	Targeted to youth with a high risk driving or alcohol prior drug-related police charge
Nores 2005 [103]	High/Scope Perry	High risk children
Palinkas et al. 1996 [104]	Social skills training	Pregnant and non-pregnant (at risk for pregnancy) adolescents using/at risk of using drugs
Pantin 2009 [105]	Familias Unidas	Hispanic adolescents in grade 8 with behavioural problems, and their primary caregivers
Redding 2015 [106]	Step-by-Step	Females, aged 14–17 years, not pregnant
Sanchez 2007 [107]	Reconnecting youth	Targeted to persons experimenting with drugs or other risk-related behaviours
Schwinn 2010 [108]	RealTeen	Targeted to girls living in public subsidized housing
Shetgiri 2011 [109]	Shetgiri Study	High-risk status based on: rate of absence of 80% and above, 2 or more disciplinary actions in 8th grade, failing two or more classes in 8th grade, or high levels of family dysfunction identified by grade teacher, using proxies such as multiple family moves in grade 8, perceived lack of parental involvement or family conflict
Sussman et al. 1998 [110]	Towards no drug abuse - School-as-community	Continuation high school youth at high risk of drug use
Tierney 1995 [111]	Big Brothers Big Sisters	Youth from single parent households recruited for mentoring
Vitaro et al. 1996 [112]	SAPP	Young boys and girls with behaviour problems diagnosed in sample
Wagner 2014 [113]	Guided Self Change	High risk behaviour related to alcohol and violence

## Appendix 2

**Table 3:** Excluded universal studies. The universal studies excluded from the subgroup analysis and reasons for exclusion

Primary Authors	Trial	Reason for exclusion
Bauman et al. 2001 [114]	Family Matters	SES not stated
Beets 2009 [115]	Positive Action (Hawaii)	SES not stated
Bonds 2010 [116]	New Beginnings	Sample almost exclusively white middle class.
Clayton et al. 1996 [117]	DARE	SES not stated
Dent et al. 2001 [118]	Towards no drug abuse - General high schools	SES not stated
Dolan 2010 [119]	BBBS Ireland	SES not stated
Donaldson 1994 [120]	AAPT	SES not stated
Eisen et al. 2003 [121]	Lions quest	SES not stated
Fearnow-Kenney 2003 [122]	All stars Sr	SES not stated
Furr-Holden et al. 2004 [79]	Family-schools partnership	SES not stated
Griffin 2006 [123]	Life Skills Training	Participants primarily from middle class suburban background
Hansen [124]	AAPT	SES not stated
Li 2011 [125]	Positive Action (Chicago)	SES not stated
McCambridge et al. 2005 [126]	Motivational interviewing 2005	SES not stated
McCambridge et al. 2008 [127]	Motivational interviewing 2008	SES not stated
McNeal 2004 [128]	All Stars 1	SES not stated
Melnyk 2013 [129]	COPE	SES not stated
Moskowitz et al. 1984 [130]	Napa	Predominantly white, middle-class and suburban community
Nader 1999 [131]	CATCH 3	SES not stated
Olds 1998 [132]	Nurse Family Partnership 1	Participants primarily low socioeconomic, unmarried mothers
Palmer [133]	AAPT	SES not stated
Patton 2006 [134]	Gatehouse Project	SES not stated
Pentz et al. 1989 [135]	Midwestern Prevention Project - Indiana	SES not stated
Perry 2003 [136]	DARE v DARE+	SES not stated
Roberts et al. 2011 [137]	Aussie optimism	SES not stated
Saraf 2015 [138]	Saraf	SES not stated
Schaps et al. 1982 [139]	Drug education course	Predominantly white, middle-class and suburban community
Schinke et al. 2009 [140]	Mother-Daughter - Non-Specific Population	SES not stated
Shek 2011 [141]	PATHS	SES not stated
Simons-Morton 2005 [142]	Going Places	SES not stated
Sloboda 2008 [143]	ASAP	SES not stated
Sun et al. 2006 [144]	Towards no drug abuse - Self-instruction	SES not stated
Sun et al. 2008 [145]	Towards no drug abuse - TND-4	SES not stated
Taylor 2000 [146]	AAPT	SES not stated
Valente et al. 2007 [147]	Towards no drug abuse - TND Network	SES not stated
Walter 1986 [148]	Know Your Body 1	SES not stated

## Abbreviations

CI: Confidence Interval
FAS: Family Affluence Scale
HBSC: Health Behaviour in School-Aged Children
MRB: Multiple Risk Behaviour
N: Number
PRISMA: Preferred Reporting Items for Systematic Reviews and Meta-Analyses
RR: Relative Risk
SES: Socioeconomic Status
